# The Microbiota–Gut–Brain Axis and Alzheimer’s Disease: Neuroinflammation Is to Blame?

**DOI:** 10.3390/nu13010037

**Published:** 2020-12-24

**Authors:** Ashwinipriyadarshini Megur, Daiva Baltriukienė, Virginija Bukelskienė, Aurelijus Burokas

**Affiliations:** Department of Biological Models, Institute of Biochemistry, Life Sciences Center, Vilnius University, Sauletekio Ave. 7, LT-10257 Vilnius, Lithuania; avee.megur@gmail.com (A.M.); daiva.baltriukiene@bchi.vu.lt (D.B.); virginija.bukelskiene@bchi.vu.lt (V.B.)

**Keywords:** microbiota, Alzheimer’s disease, microbiota–gut–brain axis, neuroinflammation, probiotics

## Abstract

For years, it has been reported that Alzheimer’s disease (AD) is the most common cause of dementia. Various external and internal factors may contribute to the early onset of AD. This review highlights a contribution of the disturbances in the microbiota–gut–brain (MGB) axis to the development of AD. Alteration in the gut microbiota composition is determined by increase in the permeability of the gut barrier and immune cell activation, leading to impairment in the blood–brain barrier function that promotes neuroinflammation, neuronal loss, neural injury, and ultimately AD. Numerous studies have shown that the gut microbiota plays a crucial role in brain function and changes in the behavior of individuals and the formation of bacterial amyloids. Lipopolysaccharides and bacterial amyloids synthesized by the gut microbiota can trigger the immune cells residing in the brain and can activate the immune response leading to neuroinflammation. Growing experimental and clinical data indicate the prominent role of gut dysbiosis and microbiota–host interactions in AD. Modulation of the gut microbiota with antibiotics or probiotic supplementation may create new preventive and therapeutic options in AD. Accumulating evidences affirm that research on MGB involvement in AD is necessary for new treatment targets and therapies for AD.

## 1. Introduction

Dementia is a non-curable syndrome which over time leads to a progressive decrease in memory, thinking, and the capacity to perform everyday activities [1]. There are alternative forms of dementia which include vascular dementia, dementia with Lewy bodies, and frontotemporal dementia [2], which can be provoked by neurodegenerative disorders, cerebrovascular disease, brain injury [3], and infections [4]. The progression of dementia can result in a lack of consequential speech generation and inability to understand scriptural as well as phonetic language, failure to recognize and identify objects, execution of poor motor skills, and incapability to think abstractly and to execute paradoxical tasks [4,5].

Alzheimer’s Disease (AD) is a persistent neurodegenerative (neuronal loss) disorder [6,7] which was first described by Alois Alzheimer in 1906 [8,9] while investigating a female patient Auguste Deter [10]. AD is known to be the major cause of dementia worldwide, mainly observed in the elderly [11], accounting for approximately 60–70% of all dementia cases [12]. The incidence of AD is higher in women than in men. AD is an extremely incapacitating disorder, progressing from slight memory impairments to a complete loss of mental function, and in the long period, resulting in death [13]. AD can affect distinct people in various ways. Most of the common warning signs include depression [14], memory loss, challenge in planning a task and problem-solving skills, confusion in recognizing time, mood swings and personality shifts, poor judgment in motor activities, difficulty in memorizing the literature, etc. [15].

Many factors can contribute to AD, but the greatest risk factors are determined to be exacerbations due to aging [16,17,18], degradation of anatomical pathways [12], environmental factors [19,20,21], mitochondrial dysfunction [22,23], immune system dysfunction [24,25], and genetic factors including mutations of amyloid precursor proteins (APP) [26,27].

In this review, we will be focusing on the role of the gut microbiota on the brain. We will be discussing the recent findings which show that a disturbance in the microbiota-brain axis can lead to neuroinflammation giving rise to AD. We will be discussing the recent studies which draw attention towards neuroinflammation in the brain, eventually leading to neuronal loss. Finally, we will be focusing on the administration of antibiotics and pre-vand probiotics modulating the brain function and used as a therapeutic agent in curing AD.

## 2. AD Pathology

The two major markers contributing to AD progression include amyloid-beta (Aβ) plaques and neurofibrillary tangles (NFTs) [28,29]. It was proposed that Aβ plaques are developed originally in the orbitofrontal, basal, and temporal neocortex regions of the human brain [30,31]. The accumulation of Aβ stimulates NFT formation [32,33]. The main constituent of NFTs is the protein tau in a hyperphosphorylated form. It is a highly soluble protein playing an essential role in maintenance of the stability of microtubules in the axons of neurons [34]. NFTs formed inside the neuron disrupt the microtubule structure and form an insoluble substance, which is detected in the locus coeruleus, and transentorhinal and entorhinal areas of the brain [35]. In the curtailed stage, it can spread to the hippocampus and neocortex [36]. The aggregation of plaques and tangles is followed by microglia recruitment surrounding the plaques [37]. This raises microglial activation and local inflammatory response which advance the neurotoxicity [25]. Aβ has been recognized as an antimicrobial peptide that activates the immune pathways recognized by toll-like receptor 2 (TLR2) leading to neuroinflammation [38].

**Figure 1:**
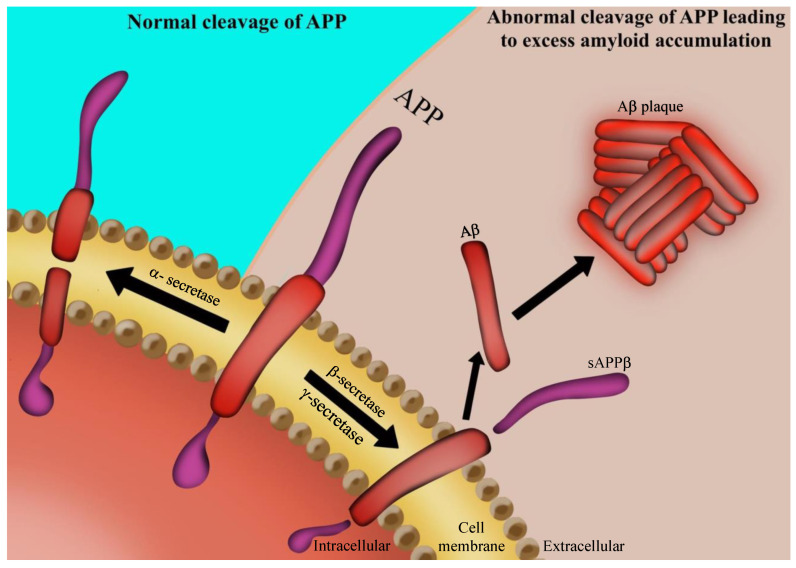
Aβ formation: the amyloid precursor protein (APP) is a transmembrane protein of the neuronal cell. In the case when it is cleaved by α-secretase, the formed soluble aggregates can be digested by microglial cells. When APP is cleaved by β-secretase and γ-secretase, it leads to formation of Aβ insoluble aggregates. Such protein aggregation results in amyloid plaques, one of the hallmarks of AD.

A recent study has shown that amyloid pathogenesis begins with altered cleavage of APP β-secretase and γ-secretase to produce insoluble Aβ fibrils [22,39] (Figure 1). Aβ then oligomerizes, diffuses into synaptic clefts, and interferes with synaptic signaling [40]. Subsequently, it polymerizes into insoluble amyloid fibrils that aggregate into plaques [31]. This polymerization leads to activation of kinases [30], which can accelerate hyperphosphorylation of the microtubule-associated tau protein and its polymerization into insoluble NFTs [41].

NFTs are fragments of paired and helically wound protein filaments in the cell cytoplasm of neurons [42]. It has the proficiency of stabilizing microtubules and forging interconnections between adjoining microtubules to form a substantial network of microtubules and to hold them together [43]. The hyperphosphorylation of tau protein occurs when it comes into contact with the kinases released due to their abundance in the environment [44]. Its hyperphosphorylation leads to the formation of oligomers [45]. The microtubule becomes highly unstable due to the dissociation of tubule subunits [46] that fall apart and then get converted into enormous chunks of tau filaments, which further aggregate into NFTs [40]. The appearance of NFTs are straight, fibrillary, and highly insoluble patches [27] in the neuronal cytoplasm [47]. The major property known causes an abnormal loss of communication between neurons and signal processing and finally apoptosis of neurons [32]. Phosphorylation of tau is regulated by several kinases, including glycogen synthase kinase-3 (GSK3) and cyclin-dependent kinase 5 activated by extracellular Aβ [48]. Even GSK3 beta and cell division protein kinase 5 are primarily responsible kinases for tau hyperphosphorylation [13], and other kinases like protein kinase C, protein kinase A [49], ERK2, serine/threonine kinase, caspase 3, and caspase 9 also have a prominent role, which may be activated by Aβ [50].

## 3. The Microbiota–Gut–Brain Axis

**Figure 2:**
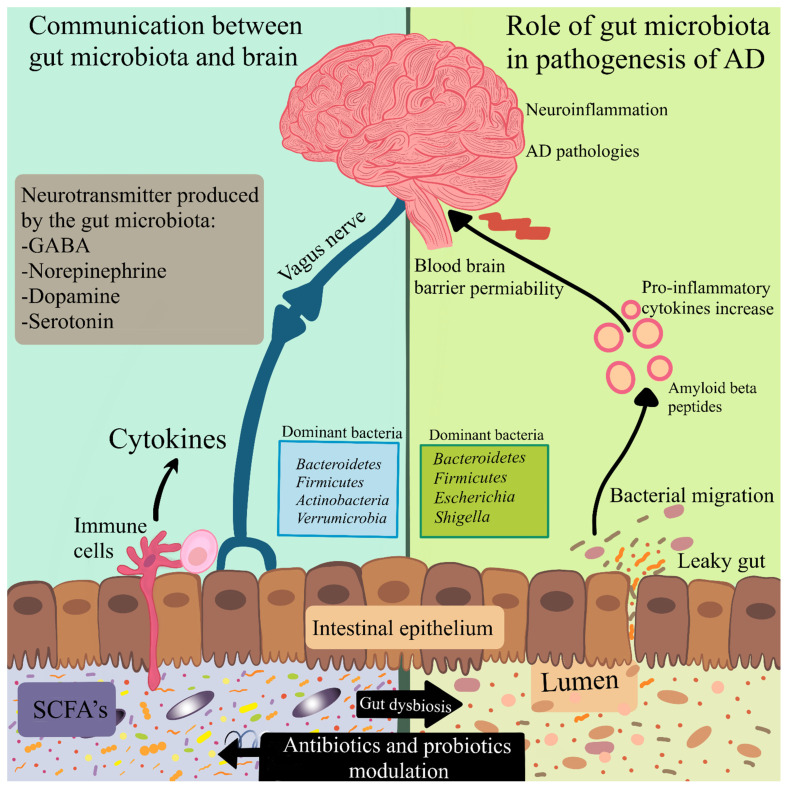
Modulation of the microbiota–gut–brain axis by antibiotics and probiotics. The communication between the gut microbiota and the brain includes neuronal, immune-mediated, and metabolite-mediated pathways. Gut dysbiosis leads to activation of the immune response and alters the production of neurotransmitters as well as bacterial metabolites. These may have a contribution to abnormal signaling through the vagus nerve. Reduction in the integrity of the gastrointestinal barrier causes bacterial migration and inflammation. Pro-inflammatory cytokines induce disruption of the blood–brain barrier permeability. Antibiotics can hinder the growth of certain bacteria, and probiotics have the potential to normalize the gut microbiota in microbiota–gut–brain processes.

A microbiota is an ecological community of commensal microorganisms that live symbiotically and pathogenically in our body [5] and plays a vital role in regulatory functions in health and disease [51,52] (Figure 2). At the level of bacterial strains, the gut microbiota demonstrates tremendous diversity and variation in microorganisms related to the age of the person and can be different in the individuals [53]. To date, it was considered that microbial colonization in the gut was only involved in colon-specific activities, which includes fermentation of carbohydrates, vitamin synthesis, and metabolism of xenobiotics [54,55]. Furthermore, it was also found that the role of the gut microbiota is to act as a barrier for the pathogenic bacteria invading the gastrointestinal tract (GIT) [56].

The microbial colonization in humans is estimated to begin at birth. The new born infant is initially colonized by microorganisms common to its mother, which are *Lactobacillus* and *Prevotella* spp. [57]. When compared with healthy and preterm infants, usually delivered by caesarean section, preterm infants seem to have variations in the microbiota [58]. As well, further comparison with elderly people in nursing homes and in the community showed large differences. The individuals in the nursing home had less microbiota attributed to a limited diet [59]. Alterations of the composition of microorganisms due to dietary changes can result in augmentation of several diseases such as obesity, colorectal cancer, inflammatory bowel disease, heart failure, type 2 diabetes, and neurodegenerative disorders (AD, Parkinson’s disease, multiple sclerosis, etc.) [52,57,60,61]. Furthermore, antibiotic treatment in early life can modulate the composition of microbiota in the gut later in life and can have a negative impact on the brain functions [62,63].

Numerous studies indicate that gut microbiota can have an influence in synthesizing various neurotransmitters and neuromodulators, which affect gut–brain communication and brain function [64,65,66]. Signal transduction is complex and can have the propensity to include neural, endocrine, immune, and metabolic pathways. However, its detailed mechanism and signals still have to be elucidated [53,67,68]. Clinical and preclinical studies have shown that gut microorganisms can produce metabolites, which affect brain functioning (Table 1).

Bacterial strains such as *Escherichia*, *Lactobacillus*, *Saccharomyces*, and *Bacillus* can synthesize amino acids including gamma-aminobutyric acid, 5-hydroxytryptamine, dopamine, butyrate, histamine, and serotonin, which can play a significant role in emphasizing the brain activity of the individuals [84,85]. These neurotransmitters synthesized can cross the mucosal layer of the intestine and are capable of entering the blood stream [61,86]. It was found that the microbiota of aged individuals with AD have a lower level of bacteria that resulted in decreased butyrate levels [87], which, in turn, could lead to increased inflammation in the brain and the progression of cognitive loss [27,86]. These findings suggest that the microbiota performs numerous vital functions in our body, including releasing biochemical by-products such as SCFA and gases [88]. Moreover, animal studies conducted on pigs and rats showed an effect on memory due to microbiota, *bacillus* and *saccharomyces* [85,86,87]. Interestingly, a recent study has shown that microbiota transfer from human subjects with obesity led to reduced memory scores in mice, aligning this trait in humans with that of recipient mice [89], where RNA sequencing of the medial prefrontal cortex of those mice uncovered that short-term memory is associated with aromatic amino acid pathways, inflammatory genes, and clusters of bacterial species [89].

As the GIT of humans are inhabited by numerous microorganisms essential for by-product formation, it has been recently reevaluated in functional terms and different important mechanisms have been established in the bidirectional connection with the brain [90,91,92]. This bidirectional connection with the brain is termed as the “microbiota–gut–brain (MGB) axis”. MGB refers to a crosstalk between the brain and the gut involving multiple overlapping pathways, including the autonomic, neuroendocrine, vagus nerve, the immune system, or the metabolic processes of gut microorganisms and immune system as well as bacterial metabolites and neuromodulatory molecules [93,94]. The MGB axis mirrors the constant connection between the central nervous system (CNS) and the GIT [95]. A number of rodent studies suggest potential involvement of the gut microbiota in behavioral changes [75,96,97,98]. The sympathetic and parasympathetic arms of the autonomic nervous system, including the neuroendocrine and neuroimmune systems, are known to be vital pathways in MGB [99]. The precise mechanism that arbitrates gut–brain interplay is not fully comprehended, yet it is suggested that it entails immune, endocrine, and neural pathways, leading to a possible alteration in AD patients or aggravation of inflammation (Table 2). The results from a rat study showed that *Bifidobacterium infantis*, an intestinal resident microorganism, has a link to immune response in the brain [75]. An augmentation in the number of *Lactobacillus casei*, *Bacteroides fragilis*, and *Streptococcus thermophilus* in the rodent intestine showed a positive effect on brain activity and performance [75,98,99,100,101,102]. On the other hand, *Eubacterium rectale*, *Porphyromonas gingivalis*, and *Lactobacillus rhamnosus* can play a vital role in the onset of AD [103,104,105,106,107].

Consideration of the human microbiota as a substantial correspondent to nutrition, health, and disease is a relatively fairly contemporary study, and currently, peer-reviewed studies relating modifications in the microbiota to the etiopathology of human diseases are few [108]. Claims on the potential involvement of the gut microbiota in brain function are made, in part, due to the well-described pathways of communication between the brain and the GIT which has been intensively studied in the area of food intake, satiety, and regulation of the digestive tract [109].

Incorporation of certain microorganisms, such as probiotics, in diet intake can be used as a therapeutic strategy to reduce neurological disorders. *Bifidobacterium* and *Lactobacillus casei* are two microorganisms which show a beneficial effect on neurological disorders [75,112].

## 4. Gut Microbiota in AD

Changes altering the gut microbiota can activate proinflammatory cytokines and increase intestinal permeability, which lead to the development of insulin resistance that is associated with AD [117] (Figure 2). Interestingly, recent work has shown that AD development could start even in the gut and then spread to the brain [118]. In this study, the gastric wall of mice was injected with Aβ_1–42_ oligomers. Over 1 year, it was observed that the amyloid migrated from the intestine to the brain. Consequently, the translocation of Aβ oligomers from the gut to the brain can have a major contribution in causing AD and neuroinflammation [118].

*Escherichia coli*, *Salmonella enterica*, *Bacillus subtilis*, *Mycobacterium Tuberculosis*, and *Staphylococcus aureus* are some of the bacterial strains that can produce functional extracellular amyloid fibers [107]. These amyloid proteins help the bacterial strains to form biofilms and to strongly bind to each other to resist destruction by physical and immune factors [119]. The amyloids formed by bacteria are different from the CNS amyloids in the primary structure but show resemblance in their tertiary structure [120]. The appearance of bacterial amyloid in the gut can trigger the immune system, which could lead to enhanced immune responses with endogenous formation of neuronal amyloid in the brain [119]. Studies of AD patient’s blood and cerebrospinal fluid showed an escalated inflammatory response when compared to healthy adults [107]. In the latter case, the clearance of amyloid is very precise [121].

In a recent study, aged Fischer 344 rats were orally exposed to transgenic *E. coli* producing the extracellular bacterial amyloid protein curli (a type of amyloid fiber protein). The data showed an enhanced alpha-synuclein production in the gut and intensified aggregation of alpha-synuclein in the brain, leading to enhanced microgliosis and astrogliosis. Elevated expressions of TLR2, IL-6, and TNF-α in the brain of animals exposed to curli-producing bacteria were determined. This suggested that bacterial amyloid functions as a trigger initiating alpha-synuclein aggregation through cross-seeding and prime responses of the innate immune system [122].

A profound experiment conducted on the APP transgenic mouse model for AD suggested that variation in the number of microbial strains could lead to amyloid deposition. These APPPS1 mice showed reduced numbers of *Firmicutes* and an increased number of *Bacteroides* in the intestine. The germ-free APP transgenic mice demonstrated a reduction in cerebral Aβ pathology [123]. This finding strongly points towards the intestinal microbiota forming amyloid-triggering immune responses that can lead to hallmarks of AD.

Clinical studies of the gut microbiota of AD patients as well as microbiota from AD model mice revealed decreased microbial diversity when compared with controls (Table 3). These include decreased levels of *Fusobacteriaceae*, *Firmicutes*, *Actinobacteria*, and *Bifidobacterium* and increased levels of *Bacteroidetes* [54,124]. *Cyanobacteria,* one of the gut-residing bacteria, produces a neurotoxin β-N-methylamino-L-alanine, which interferes with the N-methyl-D-aspartate glutamate receptor and leads to signal dysfunction in AD [125].

Not only the bacterial strains residing in the gut can lead to neurodegeneration but also the invading pathogens, such as *Mycobacterium leprae,* are known to be responsible for demyelination and nerve damage. *M. leprae* assists in initiation of the pathogen by changing the internal environment of Schwann cells and stimulation of apoptotic pathways in cells [131]. *Chlamydia pneumoniae* causing respiratory tract infection has been reported in CNS disorders, including AD [132]. *C. pneumoniae* antigens were also found in the neocortex of AD in association with NFTs and senile plaques [133]. Moreover, *Cladosporium*, *Malassezia*, *Phoma*, *Saccharomyces,* and *Candida* species DNA, polysaccharide, and proteins were observed in the CNS samples of AD patients [134]. Fungal footprints were identified in the cerebrospinal fluid by using PCR and slot bolt assay techniques [135].

Upon infection, various cell signaling pathways can occur in the body, which can activate inflammation. When infectious microorganisms cross the blood–brain barrier, it leads to neuronal death due to inflammation and forms similar hallmarks to AD. Lipopolysaccharide (LPS) is found in many gram-negative bacteria [136], exclusively on the outer membrane [137]. An experiment conducted on animal models has shown that bacterial LPS injection in the fourth ventricle of the brain produced inflammatory and pathological characteristics as observed in AD [138] and the peritoneal cavity led to extended elevation of Aβ in the hippocampal regions of mice resulting in cognitive decline [139]. An in vitro study conducted on *E. coli* confirmed that bacterial LPS advanced amyloid fibrillogenesis [127]. Studies conducted on AD patients confirmed LPS presence in the hippocampus and neocortex brain lysates in which most of the LPS aggregation has been observed in the perinuclear region [129,140]. The LPSs are located near Aβ 1-40/42 in amyloid plaques as well as blood vessels [128], and in AD patients, its levels are slightly higher compared with healthy adults [141]. When microglial cells come in contact with LPS, the TLRs present on the cell membrane of microglia gets activated through interaction with glycosylphosphatidylinositol-anchored receptor CD14 and MD-2 protein promoting inflammatory responses [110,142]. CD14-activated receptor TLR4 mediates responses to Aβ [143]. This activation affects the immune response and induces neuroinflammation.

## 5. Neuroinflammation

Our brain sustains the immune cells that protect against infection and injury, also supporting neurons in plasticity and circuit efficient connectivity. Inflammation is a response necessary for protection and regulation of the process which is associated with managing and reducing damage of the organism: protection against microorganisms, tissue repair, and removal of debris from the body [144]. Various studies currently indicate the involvement of neuroinflammation playing a crucial role in the progression of neuropathological changes that are observed in AD [145] (Figure 2). A broad variety of cellular and molecular mechanisms, assumedly identical in aging and chronic metabolic diseases such as hypertension, diabetes, metabolic syndrome, dementia, depression, or traumatic brain injury, are currently considered silent contributors to neuroinflammation [146]. The key players responsible for induction of neuroinflammation are known to be activated microglia and astrocytes [24,147].

Microglia which originate from myeloids are known as immunocompetent cells in the brain. Microglia cells are considered to be the most important player in the development and progression of neuroinflammation [25]. Microglia are immensely plastic cells that can transform into complex phenotypes depending on specific microenvironmental signals within the brain [148]. On the membrane, these cells express a diverse range of innate immune receptors that belong to the pattern recognition receptors family [147]. When pattern recognition receptors get activated on microglia, activation of the cell and the production of inflammatory mediators occur in the presence of a distinct signaling cascade [149]. Repeatedly activated microglia release a broad range of proinflammatory [150] and toxic products and, among them, reactive oxygen species, nitric oxide, and cytokines. In addition, endothelial cells and perivascular macrophages are also important in interpreting and propagating these inflammatory signals within the CNS [24]. A threat to the CNS, such as invasion, injury, or disease, activates microglia, induces morphological changes, and increases motility of cells.

In AD, there are studies conducted that the primary initiator of activation of microglia is the accumulation of Aβ [151]. The activated microglia respond to Aβ, resulting in migration to the plaques and phagocytosis of Aβ. It initiates a microglial-mediated inflammatory response by binding to various pattern recognition receptors [152], which, in turn, results in cell activation and release of proinflammatory factors (iNOS, TNF-α, IL-1, and IL-6) [152,153,154,155]. In the case of AD, the receptors present on the surface of the microglia bind to Aβ oligomers and Aβ fibrils. In the process of phagocytosis, microglia begin to clean up Aβ fibrils; hence, fibrils undergo an endolysosomal pathway.

Other than microglia, astrocytes are also major participants in neuroinflammation [156]. They are fivefold more than neurons in the CNS [157] and are known to have functions in the maintenance of CNS integrity, such as control of blood perfusion in the cerebrum, maintenance of blood–brain barrier stability, and modulation of neuron or nutrient transmission [158]. In AD patient brains, there have been observed alterations in the morphology of astrocytes, their protein composition, gene expression, and function [150]. The accumulation of activated astrocytes is often present in clusters around amyloid plaques. Aβ deposit can activate the astrocytes which lead to overexpression of cytokines, such as IL-1β and IL-6, resulting in oxidative stress [24,159]. It was recently shown that neurodegeneration presumably associates astrocytes, which, by taking on a microglia-induced A1 proinflammatory phenotype, would encourage neuronal cell death, with TNF-α as the most eminent arbitrator [160,161].

On the other hand, the activated microglia lose their phagocytic effect, thus decreasing the degree of Aβ phagocytosis, inevitably developing its accumulation [162]. Moreover, such discoveries are supported by the results of an association between an increase in AD risk and alterations in genes encoding immune receptors such as TREM2, CD33, and CR1 (myeloid cell surface antigen) [163]. Since they are all expressed on myeloid cells, it is a more convincing demonstration that alterations in microglial biology are linked to AD pathogenesis. Worth mentioning, a variety of transcriptomic and proteomic analysis of inflammatory cells might provide biomarkers for preclinical detection as well as insights on the progression from mild cognitive impairment to AD condition [164,165,166].

A relatively close connection has also been reported between microglia and cognitive dysfunction [167]. Importantly, in healthy tissue, microglia have a ramified morphology and prolongations that continuously look after the synaptic activity. However, phagocytic microglia have a salient role in synaptic pruning and honing in the developing nervous system [168]. The most fascinating mechanism describing memory dysfunction in AD suggests that Aβ oligomers lead to microglial activation, which, in turn, excessively engulfs and accelerates the termination of synapses through complement factors such as C1q and C3 [169]. It has also been reported that Aβ oligomer arbitrates memory problems which are closely connected with glial activation [100,170].

Recent evidences now shed light on a dangerous dialogue between central immune cells and the gut microbiota, potentially leading to AD in humans.

## 6. The Link between Microbiota and Neuroinflammation

The immune system modulates the gut microbiota framework and issuance [171], while in return, the microbial symbionts control immune system maturation and function [172,173]. Numerous rodent studies have affirmed that there is an interaction between the gut microbiota and various immune cell populations [174,175] or the expression of genes related to neuroinflammation [176,177].

The study furnished evidence stating that microbiota residing in the gut predisposes the development of the immune system by administering hematopoiesis of primary immune cells. It was shown that germ-free (GF) mice have a lower ratio and less distinction capability of myeloid cell progenitors of both yolk sac and bone marrow origin. This supports the idea of the widespread effects of gut microbiota on the immune system, microglia included [175]. Microglia from antibiotic-treated mice or GF mice showed an immature profile and impaired immune response. The absence of gut microbiota alters microglial mRNA profiles and suppresses various microglial genes involved in cell activation, pathogen recognition, and host defense. Microglia transcription and survival factors, normally suppressed in mature adult microglia, were increased in GF mice [178]. The experiment was conducted to examine the transcriptional profiles of different microglial development stages, referring to the genes related to the adult phase of microglial maturation and immune response that are abnormally regulated in GF mice [179].

A number of studies have coined a protective association between dietary polyphenols and the prevention of age-related chronic diseases such as diabetes, cancer, and neurodegenerative diseases [180,181,182]. Dietary flavonoids and nonsteroidal anti-inflammatory agents modulate the nuclear factor-kappa β signaling pathway and therefore are termed as a potential therapeutic target for AD [182,183,184]. Polyphenols make an impact on microbiota-related metabolism and have a potential to improve neurological health, including their ability to interact with intracellular neuronal and glial signaling, to modulate peripheral and cerebrovascular blood flow, and to reduce neuronal damage and loss induced by neurotoxins and neuroinflammation [185,186,187]. Flavonoids, a subclass of polyphenols, are more likely to combat neuronal dysfunction and toxicity by recruiting antiapoptotic pro-survival signaling pathways, increasing antioxidant gene expression and reducing Aβ pathology [182,188,189]. Flavonoids that are not absorbed in the small intestine and other sugars are then broken down by the gut microbiota into phenolic acids and other metabolites that inhibit the growth of *Ruminococcus gauvreauii*, *Bacteroides galacturonicus*, and *Lactobacillus* sp. strains [190] and flavonoids present in berries have also shown inhibitory actions against *Bacillus cereus*, *Campylobacter jejuni*, *Clostridium perfingens*, *Helicobacter pylori*, *Staphylococcus aureus*, *Staphylococcus epidermidis*, and *Candida albicans* [191]. Recently, it was reported that anthocyanins (one of the flavonoids) could significantly ameliorate the expression of proinflammatory cytokines and ROS/JNK, thus preventing neuroinflammation and AD pathology [192,193,194]. In an experiment conducted on aged rodents, blueberry supplementations have shown improved spatial memory, object recognition memory, and inhibitory fear conditioning learning [195,196,197]. In another study on blueberry anthocyanins given to adults aged 40–74 years over 3 weeks, plasma concentrations of NF-kB-related proinflammatory cytokines and chemokines (IL-4, IL-13, IL-8, and IFN-α) were significantly reduced [198]. However, a study conducted by Spilsbury et al. did not reveal any remarkable effect of lower concentrations of flavonoids on NF-κB activity in astrocytes [199]. Nevertheless, the literature date supports that the dietary supplementation of flavonoids might be implicated in the regulation of NF-κB in neurons [199].

Flavonoids are important players in the prevention of neuroinflammation via several anti-inflammatory mechanisms, inhibiting the microglial activation of inflammatory cytokines (TNF-α and IL-1β), inhibiting iNOS and ROS generation in activated glia, and downregulating the activity of pro- inflammatory transcription factors such as NF-κB through modulation of glial and neuronal signaling pathways [182].

Chicory root, known for its high content of fibers (galacto-oligosaccharides and fructans, such as inulin) and beneficial for the MGB axis modulation [64,177,200], recently also has received attention due to its sesquiterpene lactones (a class of sesquiterpenoids that contain a lactone ring) [201]. Interestingly, it has been shown that different sesquiterpene lactones from chicory root have the potential to influence anti-inflammatory responses through modulation of the nuclear factor of the activated T-cells pathway [201].

Bacterial metabolites such as SCFAs were considered the key mediators for microbiota–microglia interaction. These compounds have the potential to translocate from the mucosa to systemic circulation and to cross the blood–brain barrier affecting the CNS and their function [68,202]. Oral administration of SCFA for 4 weeks restored many facets of the immature microglial morphology of GF mice. SCFA claimed to reestablish microglial density and normalized CSF1R surface expression [203]. It is crucial to accentuate that the gut microbiota–microglia interaction is extremely dynamic as many of the defects noticed in the microglia of GF mice could be partially restored by recolonization with conventional gut microbiota or SCFA supplementation [203].

## 7. Role of Antibiotics on Microbiota in AD

Antibiotics or antimicrobial substances are typically used to remove or prevent bacterial colonization in the human body [204]. These can alter the bacteria without any specific target or type [205]. As a consequence, a broad spectrum of antibiotics can immensely affect the composition of the gut microbiota, lower its biodiversity, and withhold colonization for a long period after administration. Various studies with distinct antibiotic treatments resulted in long-/or short-term changes in the gut microbiota in both animals as well as humans [206]. Numerous studies have demonstrated that the use of antibiotics has an association with changes in behavior and brain chemistry [207,208,209]. Studies conducted in vivo with long-term broad spectrum antibiotic treatment have shown a decreased Aβ plaque deposition, attenuation of plaque localization in glial reactivity, and alteration in microglial morphology in the APP_SWE_/PS1_ΔE9_ mouse model of AD [210]. Another study conducted on 68 patients with advanced AD demonstrated a correlation among usage of antibiotics and prolonged survival. Of the patients who survived for more than 6 months, 31% were on antibiotic care and 14% were on palliative care [211]. Another study in humans showed that antibiotics, i.e., cefepime, can cross the blood–brain barrier, causing altered mental status, along altered consciousness and confusion without mediation of the gut microbiota [212]. Below, some of the preclinical studies of antibiotics in animals and humans have been described briefly.

The patients suffering from infection caused by *Helicobacter pylori* were administered with a cocktail of antibiotics consisting of proton pump inhibitor and clarithromycin, along with amoxicillin or metronidazole. The outcome of this treatment showed an association with neurological disorders, including panic attacks due to major depression and anxiety, delirium, and psychosis [213]. On the other hand, the elimination of pathogenic bacteria such as *Helicobacter pylori* in AD patients by the triple eradication antibiotic regimen (clarithromycin, amoxicillin, and omeprazole) led to positive results for cognitive and functional status parameters [214].

Antibiotic administration with rifampicin and minocycline in AD animal models reduced the Aβ levels in the brain and abbreviates inflammation cytokines [215]. Oral administration of rifampicin to three different mouse models of Alzheimer’s disease and tauopathy showed that this antibiotic reduced the accumulation of Aβ oligomers and tau oligomers and enhanced the memory of the mice. These results suggested that rifampicin could prevent AD [216]. In 6 months, AD patients’ improvement in the Standardized AD Assessment Scale cognitive subscale was observed when treated with a combination of doxycycline and rifampicin [217].

A pilot study conducted on the TgCRND8 transgenic mouse model showed that 3 months of treatment with erythromycin in drinking water at 0.1 g/L reduced the Aβ_1-42_ levels in the cortex by 54% when compared to vehicle-treated mice [218].

Several studies conducted on minocycline suggested that it has neuroprotective and anti-inflammatory actions in many animal models. In microglial cell cultures, it was remarkably able to reduce the oligomeric Aβ-induced neuroinflammatory response and enhancement of fibrillar Aβ phagocytosis [219]. Minocycline treatment at 50 mg/kg for 4 weeks in a transgenic hAPP mouse model of AD exhibited attenuated behavioral abnormalities, neuroinflammatory markers, and Aβ [220]. In another study, 4 months of treatment with minocycline at 55 mg/kg/day in food in 3×Tg-AD mice showed a reduction in brain levels of insoluble Aβ, decreased neuroinflammatory markers, and reversed cognitive deficit [221].

A contrary effect of antibiotics was also observed after administration of ampicillin in the Sprague–Dawley rats. In this case, an elevated level of corticosterone in serum, intensified anxiety-like behavior, impairment of memory due to elevated glucocorticoids, and reduction in hippocampal brain-derived neurotrophic factor were determined [222]. Distinct studies demonstrated that administration of intracerebroventricular streptozotocin into the brain of wild-type mice and rats can cause learning impairment and memory loss [223,224,225,226,227].

An experiment conducted on APP_SWE_/PS1_ΔE9_ transgenic mice administered with antibiotics demonstrated that it led to an alteration in several circulating inflammatory cytokines and chemokines in the blood. It also showed an elevated level of CCL11 (which has a link to age-related deficits in hippocampal neurogenesis) [228] in the blood serum of mice [210]. A recent study conducted on APP_SWE_/PS1_L166P_ mice treated with a cocktail of antibiotics revealed a selective, microbiome-dependent, sex-specific effect on brain Aβ amyloidosis of Aβ and microglial physiology [229]. Interestingly, the transplants of fecal microbiota from age-matched APP_SWE_/PS1_L166P_ mice into antibiotic-treated APP_SWE_/PS1_L166P_ mice restores the gut microbiota and partially restores AD pathology along with microglial morphology [229].

## 8. Role of Probiotics on Microbiota in AD

Probiotics are defined as living microbial feed supplements which show a beneficial effect on the host, resulting in improved intestinal microbial balance [230]. The most commonly used probiotics are lactic acid bacteria, particularly *Lactobacilli*, *Streptococci*, *Pediococcus*, *Enterococcus*, and *Bifidobacteria* and some yeast like *Saccharomyces boulardii*. However, not all microorganisms can be probiotic, as they need to be strain-specific (Table 4).

A broad range of probiotics have been used in an animal study and in the models of AD. In rats, *Bifidobacterium* and *Lactobacillus* administration have shown a positive effect on AD treatment [235]. In an AD mouse model, *Bifidobacterium breve* strain A1 prevented cognitive function, making it one of the effective treatments for AD [237]. A reduction in neuroinflammation in mouse models due to *Lactobacillus casei* strain *Shirota* can be effective against AD [234]. Despite the fact that there are few human clinical studies compared to animals, there is increasing indication that probiotics can be used for reducing depression and anxiety-like symptoms [241].

A study with thirty-six healthy women assigned to three groups showed the importance of probiotics in the modulation of brain activity [242]. In this experiment, the group which was treated with fermented milk products containing *Bifidobacterium animalis sub. lactis*, *Streptococcus thermophilus*, *Lactobacillus bulgarigaricus*, and *Lactococcus lactis subs. lactis* showed a compelling reduction in the activity of the specific area in the brain. This region of the brain is involved in sensory/affective tasks when compared to the activation of other cortical regulatory brain areas. The experiment confirmed that probiotic supplementation has a major contribution in activating specific areas in the brain involved in the central control of emotion and sensation [242].

In another study conducted to understand the probiotic application in AD, sixty patients with AD were randomly assigned into two groups [243]. The first group received 200 mL/day milk enriched with *Lactobacillus acidophilus*, *Lactobacillus casei*, *Bifidobacterium bifidum,* and *Lactobacillus fermentum* for weeks, whereas the control group received plain milk of the same amount. The subjects, which were on probiotic supplementation showed a significant improvement in the mini-mental state examination test when compared with controls. The study revealed a beneficial effect on cognitive function and metabolic status of patients with AD. However, the treatment with probiotics was ineffective on oxidative stress and inflammation [243].

A study conducted by Leblhuber et al. showed an increased level of serum kynurenine, which was observed after probiotic administration, potentially caused by macrophage activation. The stimulation of immune cells could induce mechanisms that can be helpful in removing amyloid aggregates and damaged cells or on the other perspective. On the other hand, the intensive activating events could negatively affect gut barrier function and further stimulate neurodegenerative events [244].

When taken together, these human and animal studies prove that probiotics can have a major role in the bidirectional communication between the gut microbiota and the brain, modulating brain function. The exact mechanism of probiotics on the MGB axis is not yet well defined. Therefore, the data suggest that the proper dose of probiotics in AD treatment would be a new way to eliminate amyloid deposition in the brain by the MGB axis and to reduce neuroinflammation (Figure 2).

## 9. Conclusions

Accumulating all information from the human as well as animal studies, it can be suggested that GIT microbiota has an important role in the bidirectional communication between the brain and the gut. There is increasing evidence stating that the gut microbiota has a contribution to the pathogenesis of AD. As the gut microbiota is known as the source of a large number of amyloid, LPS, and other toxins, it can contribute to systemic inflammation and disruption of physiological barriers. The products formed by bacteria can move from the GIT to the CNS, especially in aging. Bacterial amyloid can trigger misfolding and can enhance native amyloid aggregation. The gut microbiota products can activate microglia, augmenting inflammatory response in the CNS, which in turn results in microglial function. Triggered microglia start neuroinflammation in the brain, causing loss of neurons, a major factor in AD. Modulation of the gut microbiota composition can be used as a therapeutic target in AD. Some antibiotics as well as probiotics can be implemented as a preventive measure that successfully targets ongoing inflammation. The role of antibiotics and probiotics in modulating the microbiota is under intense debate. The certain microbiota profile also strongly depends on the host’s genetics and diet. This only confirms that research on MGB involvement in AD is crucial for new treatment targets and therapies for AD.

## Figures and Tables

**Table 1:** Effect of metabolites on brain produced by gut microbiota.

No.	Gut Microorganisms	Metabolites	Effects of Metabolites on Brain	Subjects	References
1	*Lactobacillus*	Short chain fatty acids (SCFA), Serotonin, Acetylcholine	Increases emotional level	Wistar rats	[69,70]
			Improves attention, memory and motivation	Humans	[71]
			Improves sleep	C57BL/6J mice	[72]
2	*Bifidobacterium*	Gamma-aminobutyric acid	Reduces anxiety, stress, and fear Improves ADHD	Humans	[69,73,74]
		Tryptophan	Improves behaviors relevant to depression	Pregnant Sprague–Dawley dams, rats	[75]
3	*Escherichia*	Dopamine, Norepinephrine, Endotoxin and Serotonin	Improves mood, blood flow, sleep regulation, cognition and concentration, hormonal activity	Human	[76,77,78,79]
4	*Bacillus*	Tryptophan	Improves cognitive function	Pigs	[80,81,82]
5	*Saccharomyces*	Norepinephrine	Enhances formation of retrieval of memory	Wistar rats	[77]
6	*Enterococcus*	Histamine, Serotonin	Promotes wakefulness, cognition orchestrates desperate behavior	C57BL/6J	[83]

**Table 2:** Roles played by different microorganisms residing in the gut.

No.	Organism	Positive ↑/Negative ↓ Effects	Subjects	Role	Reference
1.	*Bacteroides fragilis*	↑	AD patients	Protected against CNS demyelinating disease	[100,101]
			C57BL/6 mice	In pregnant mice showed an immediate significant diminished autistic behavior	[102,110,111]
2.	*Lactobacillus casei*	↑	SAMP8 mice	A decreased in anxiety symptoms	[112]
3.	*Lactobacillus rhamnosus*	↑	Wistar rats	Ameliorated the inflammation level in the brain	[103]
4.	*Streptococcus thermophilus*	↑	SJL/J mice	Robust effects on brain regions that control the central processing of emotions and sensation Degradation of Aβ 42 load	[113,114]
5.	*Bifidobacterium infantis*	↑	Sprague–Dawley dams rats	Normalized the immune response	[75]
6.	*Campylobacter jejuni*	↓	AD patients	Induced anxiety-like behavior Impaired memory	[104]
7.	*Campylobacter rodentium*	↓	C57BL/6 mice	Led to stress and contributed to behavioral abnormalities	[105]
8.	*Porphyromonas gingivalis*	↓	AD patients	Caused an inflammatory response in the liver, which subsequently led to neuroinflammation and causes neurodegenerative disease	[106]
9.	*Eubacterium rectale*	↓	AD patients	Leads to amyloidosis	[107]
10.	*Lactobacillus acidophilus*	↑	SAMP8 mice	Improved the impairment in neural proteolysis	[112,113]
11.	*Lactobacillus johnsonii*	↑	BB-DR rats Healthy humans	Improved gastric vagus nerve activity	[115,116]

**Table 3:** Investigation of microbiota in the gut of human as well as animal models of AD.

No.	Microorganisms	Increase ↑/Decrease ↓	Animal Model	Location	Reference
1.	*Firmicutes/Actinobacteria*	↓	CONVR-APP/PS1	Intestine	[54,124]
2.	*Bacteroides/tenericutes*	↑			
3.	*E. coli/B. subtilis*	↑	AD patient	Brain tissues/Stool	[69,126,127,128]
4.	*E. rectale*	↓	AD patient	Stool	[107,129]
5.	*Escherichia/shigella*	↑			
6.	*B. fragilis*	↓			
7.	*Lactobacilli/Bifidobacteria*	↑	SAMP-8 mice	Intestine	[71]
8.	*Fusobacteriaceae*	↓	AD patients	Stool	[123]
9.	*Prevotellaceae*	↑		Stool	
10.	*Verrucomicrobia*	↑	APP_SWE_/PS1_ΔE9_ (PAP) transgenic mice	Stool	[130]

**Table 4:** Effects of probiotics on neurological disorders.

No.	Probiotic Supplementation	Subject	Effect	Reference
1.	*L. helveticus* *R0052*	WT mice IL-10 deficient 129/SvEv mice	Prevented from anxiety-like behavior and memory impairment	[231]
2.	*Lactobacillus plantarum MTCC 1325*	AD rat model (IP injection of D-galactosea)	Reestablished acetylcholine levels, debilitated Aβ plaque formation, and ameliorated cognitive function	[232]
3.	*L. helveticus,* *L. rhamnosus*	Streptozocin injected rats (diabetes rats)	Improved spatial memory impairment and recovered declined basic synaptic transmission	[233]
4.	*Lactobacillus casei* strain Shirota (LcS)	In vivo mouse model of EAE	Reduced neuroinflammation	[234]
5.	*Lactobacillus* and *Bifidobacterium*	AD rat model (intrahippocampal injection of Aβ)	Ameliorated memory, learning deficits, and oxidative stress	[235]
6.	*Clostridium butyricum*	Mouse model of vascular dementia	Reduced neuronal apoptosis and attenuated cognitive dysfunction and histopathological changes	[236]
7.	SLAB51 probiotic formulation	3×Tg-AD mice	Altered plasma concentration of inflammatory cytokines and gut hormones induced also a decrease in brain damage and accumulation of Aβ aggregates	[113]
8.	*Bifidobacterium breve* strain A1	AD mouse model (ICV injection of Aβ)	Blocked Aβ-induced cognitive dysfunction and suppressed Aβ-induced changes in gene expression in the hippocampus	[237]
9.	oligosaccharides from *Morinda officinalis*	APP/PS1 mice	Ameliorated brain tissue swelling and neuronal apoptosis and downregulated the expression of Aβ	[238]
10.	*Bifidobacterium longum* 1714	Healthy humans	Reduced stress and improved memory	[239]
11.	*Lactobacillus brevis* *FPA3709*	Sprague–Dawley rats	Similar effects to a generally used antidepressant drugs	[240]

## Abbreviations

AD	Alzheimer’s disease
Aβ	Amyloid-beta
NFTs	Neurofibrillary tangles
MGB	Microbiota–gut–brain
GIT	Gastrointestinal tract
CNS	Central nervous system
LPS	Lipopolysaccharides
TLR	Toll-like receptor
SCFA	Short chain fatty acids
APP	Amyloid precursor protein
